# The Conventions at Milwaukee

**Published:** 1901-09

**Authors:** 


### Editorial.

### THE CONVENTIONS AT MILWAUKEE.

There was no mistake made last year at Old Point Comfort in the selection of Milwaukee as the Western city for the meeting of the several annual conventions. The sentiment was universal among the visitors that this beautiful city by the inland sea was superior to all other places heretofore selected. There was a general feeling of dread, especially by the Eastern contingent, that the torrid heat to which they had been subjected would be more than duplicated in the West, but the continued cool breezes experienced in this city gave renewed life to all, and increased the energy of those engaged in the work of the meetings.

The members of the Association of Dental Faculties and the National Association of Dental Examiners were first on the ground, as both bodies meet in advance of the National Dental Association. These meetings opened on August 2. The writer, not having been present at the sessions of the examiners, is not able to give any idea of the number present or work accomplished.

The Faculties had a delegated representation that covered a wide extent of territory, from New England to California. There were but two colleges in membership, out of the fifty belonging to the Association, that failed to send delegates. A number of the colleges sent alternates.

This meeting was expected to decide the question of increase of time to four years in all the colleges, and the interest in this was necessarily intense, as there was some doubt as to the final result. The resolution making the time in all colleges four years, with a course of six months, offered by Dr. Brown last year, came up in regular order. An amendment was made to this by Dr. Truman increasing the course to seven months. It was soon evident that the contest would not be on the four years, but upon the length of the course. Those favoring six months were earnest in advocating their side of the question, but upon the vote being taken, to the surprise of every one, the amendment was adopted by a large majority. On the final consideration of the original motion as amended it was not only carried by a large majority, but several colleges changed their negative votes, making it practically a unanimous decision. The fact of the passage of this resolution was at once cabled to Drs. Kirk and Brophy, in attendance at the meeting of the International Committee in session at London.

This increase of time means more for dental education than would appear to those unfamiliar with the work. The vast amount of theoretical and collateral medical training that undergraduates are obliged to undergo has left a minimum amount of time for the important practical work of dentistry. When this rule of four years, with a seven months' course, is fully in force, the fourth year can, and doubtless will, be a year of close attention to practical details. This new law cannot go into effect until 1902-03, as due notice must be given in announcements. It will affect the freshmen of that year, but not those already enrolled in the higher classes. Consequently it will take five years before the colleges can begin to estimate the full effect of this change.

There was, aside from the foregoing, an unusual amount of interesting work accomplished in the Faculties in the direction of progress. There has never been a disposition to excuse the defects in colleges, or their open violations of law, notwithstanding the assertions to the contrary made by those outside of the body and unfamiliar with its methods and practice. Colleges have been repeatedly severely punished for infraction of rules. The committee which has this in charge between the sessions, known as the Ad Interim Committee, has not had heretofore full power to discipline members. A resolution was adopted changing this, giving this committee power to expel, fine, or censure any college violating rules, its decision subject to appeal to the main body. In this connection it may be interesting to our readers to note that two colleges were fined one hundred dollars each at this meeting and one publicly censured.

The question of power to expel a member came up in an unexpected way, and although not decided by the courts, it is established upon a general principle of law that an association of this kind must have a clearly defined standard, and that then the infractions of this must be positively proved before the courts will permit the expulsion of a member where property rights are involved. This was brought unpleasantly to the cognizance of the members by an injunction from one of the courts of Milwaukee restraining the Faculties Association from all action against the National Dental College of Washington, D. C. This College was placed under discipline upon the report of a committee appointed to visit it last year. The matter came up at Old Point Comfort, but was held under advisement until this meeting. In the mean time another committee visited the school and reaffirmed the decision of the previous committee. The National Dental College in both instances, at Old Point Comfort and Milwaukee, had lawyers sent from Washington to defend its interests. If obstructing action of the Faculties means success, the legal adviser of the National should be well satisfied with the outcome, for he effectually blocked all action by that Association. The Faculties at once retained counsel, but it was found that the courts had adjourned for the summer, and that it would be thirty days before argument could be made to dissolve the temporary injunction. The matter was left in his hands and in the care of a resident member in Milwaukee.

Unless some way is made to overcome this legal obstruction the power of the Faculties will be greatly lessened, but the members of this body are not disposed to have it this way, and measures will be taken that will meet the legal objections.

The National Association of Examiners, not having been served with an injunction, immediately took action against this college and struck its name from the list of recognized schools. This will be, of course, a severe blow to that school, but it invited just such action through its ill-advised procedures. The dentists concerned in educational matters are determined to raise their profession to the highest possible standard, and will not submit with patience to any obstructionists, whether they are to be found among the dental colleges or in the courts.

One of the interesting features of the Faculties' meeting was the appearance of United States Consul J. H. Worman. Our readers are familiar with his name and work in the report which he sent to the State Department at Washington and published in this journal. He has labored persistently for the past eighteen months in Germany to secure evidence against men engaged in the diploma traffic. The evidence thus accumulated, at his own expense, for the government at Washington would not aid directly in this matter, has been of great value, and will be used against these foreign criminals who have invaded our shores to establish diploma mills for their eager customers in the Old World, for they have none in the New. The Consul's statement was interesting and convincing to the majority of the members, and steps were at once taken to raise a sum of money to meet necessary legal proceedings. To effect this the colleges were subjected to an assessment. The writer, while favoring every effort to bring these people to justice and stop this traffic, was not in favor of permitting the idea to prevail that it was the duty of the Faculties Association to do this work, or that this body had degenerated into a police organization. This organization is educational and can have no other function, and while it may very properly aid in the good work, it has no place, directly or indirectly, in the criminal courts. It is further thought that the United States government could prosecute these cases, for the men thus engaged must have used the United States mails to effect their nefarious purposes. The State authorities of Illinois are in disgrace that they permit this thing to continue with the evidence of criminality clearly established. The people abroad cannot understand anything but a centralized government, and when a State fails in its duty the general government and all the States share in the universal condemnation. The writer learned from reliable sources that active steps will now be taken to bring these diploma manufacturers to justice, but not by the general government or that of the State principally involved, but through individuals. The money for this purpose will be furnished by the Faculties Association and the National Dental Association, and the individual to be the principal actor in the work is one well known to the dental profession, and one who rarely fails in anything he undertakes. There is, therefore, an excellent prospect of the jail population being increased in Chicago; and this may not be confined to the active and open criminals, for there is evidence against others high in authority in the State of Illinois.

The National Dental Association convened on August 6 with a large attendance. The constitutional arrangement requiring all matters of a business nature to be sent to the council enabled the meeting to begin at once the reading of papers and discussions thereon. While there was but little especially new presented in these, there was much that was superior to the average of former meetings. The dental profession is just beginning to see the result of a higher standard of college work in more thoroughly digested scientific essays. The practical man doubtless felt a tinge of disappointment that there was less time given to those subjects that really make dentistry, but this was not wholly neglected.

The discussions upon the several papers were not, as a rule, of a very high order. With one or two exceptions those appointed to open the discussion had not been furnished a duplicate copy of the paper in advance, and were, consequently, placed in the unpleasant position of having but an indefinite idea of the author's meaning. In one instance the entire trend of discussion took a direction entirely opposite from the ideas the essayist intended to convey. This was due to the impossibility of gathering the author's views upon a difficult subject with one hearing. This should not occur in the future, and a duplicate copy should be in the hands of the one to open the discussion some weeks in advance of the meeting.

The National, similarly to the Faculties Association, was enlivened by the presence of United States Consul Worman. His earnest pleadings for a concentrated effort to break up the fraudulent diploma business had a good and immediate effect. A committee was appointed to take the matter into consideration, to report at once. This was done, and the Association appropriated a satisfactory sum to be placed in the treasury subject to the order of the Executive Committee, and to be used to aid in breaking up the traffic in diplomas.

The address of Dr. Black as presiding officer was an able presentation of the needs of the Association. After presenting a brief history of the American and National Associations, he contended that the constitutions of both these organizations had been defective; that upon the disbandment of the American the National took up the work upon practically the same lines, and that these failed to meet present needs. He presented a careful analysis of the constitution governing the National and proposed some radical changes, especially in section work. These suggestions were given into the hands of a special committee. After due consideration, the main features of Dr. Black's amended constitution were reported upon favorably, but the consideration was postponed until the meeting next year. In the mean time the proposed amended constitution will be printed and sent to every member.

While the writer is in full agreement with Dr. Black's modifications, he would have been pleased could a more radical change have been proposed. We need a truly scientific national body. This was not true of the American nor has it been true of the National Dental Association, and yet there is an ideal standard that the writer can see outlined somewhere in the far-distant future, which is being led up to through the measures proposed. It is probably better to make progress slowly. If the amendments are adopted, they will give increased responsibility to the sections and require increased activity on the part of the chairmen.

The President advocated a change in the time of meeting. This was not inserted in his amended constitution, this being left, as heretofore, in August. It is earnestly hoped that the members will convene next year prepared to change the time to the last of May or beginning of June. Many were deterred this year from attending on account of the great heat, and in every year the Association is deprived of the services of some of its best men for the reason that they feel unwilling to sacrifice the largest portion of the time annually devoted to recreation, and no worker needs this more than the dentist. This August work has generally been a period of torture. This meeting was an exception, and the result of an invigorating atmosphere was apparent in better meetings, more vigorous discussions, and decidedly greater interest manifested by those in attendance. Let us then get away from the heated term.

Much of the success of this meeting was due to the local committee. From the beginning there was no diminution of its efforts to make all retain the pleasantest recollections of Milwaukee. Every day the conveyances were at the doors of the hotel to carry the members and those accompanying them to all parts of interest. Tally-ho rides were daily planned. A steamboat excursion on Lake Michigan was carried out, to the great pleasure of those able to be of the party. The heavy portion of this work fell to the chairman of the local committee, Dr. G. V. I. Brown, and nothing was left undone on his part to make the meetings at Milwaukee memorable and unique in the history of dental conventions.

This brief report of the work of the dental conventions cannot be closed without an allusion to the very full exhibits made by manufacturers of dental supplies. This in itself was an object-lesson. The writer was greatly impressed with it as an evidence of the progress made in dental appliances and in dental therapeutics. It seemed to accentuate more than ever the growing appreciation of dental work and dental knowledge.

The Convention had the pleasure of listening to a report of the work of the Army Examining Board from Dr. J. S. Marshall, its president. The board has, for the present, concluded its labors, although the full number of contract surgeons permitted by the bill has not been completed. Dr. Marshall has been assigned to the Presidio, California, Dr. Oliver to the Philippines, and Dr. Morgan to Cuba.

The S. S. White Dental Manufacturing Company had on exhibition the outfit given to each of the successful candidates. It is very compact and well suited for ready transportation. The chair is of light material, easily folded up, but yet quite strong and well adapted to the work. With such a complete outfit, in many respects superior to that obtainable by any young practitioner, the young army contract surgeons should be able to make a creditable showing the coming year and justify the assertions of those who advocated this measure that it was in the interest of the army that the bill should become a law, and not primarily to afford places for needy young practitioners.

Thus closed the conventions of the year 1901, and if their success is to be the gauge of progress for the future, the years yet to be are full of promise for the advancement of dentistry to a still more honorable position than it has heretofore occupied among the active professional organizations of the world.

